# Transsynaptic neural circuit mapping of ventral hippocampus motivational control systems

**DOI:** 10.1007/s00429-026-03164-y

**Published:** 2026-07-22

**Authors:** Molly E. Klug, Mugil V. Shanmugam, Haoyang Huang, Don Arnold, Joel D. Hahn, Scott E. Kanoski

**Affiliations:** 1 Human and Evolutionary Biology Section, Department of Biological Sciences, Dornsife College of Letters, Arts and Sciences, University of Southern California, 3616 Trousdale Parkway, AHF-252, Los Angeles, CA 90089-0372 USA; 2 Quantitative and Computational Biology Section, Dornsife College of Letters, Arts and Sciences, University of Southern California, Los Angeles, USA; 3 Molecular and Evolutionary Biology Section, Department of Biological Sciences, Dornsife College of Letters, Arts and Sciences, University of Southern California, Los Angeles, USA; 4 Neurobiology Section, Department of Biological Sciences, Dornsife College of Letters, Arts and Sciences, University of Southern California, Los Angeles, USA

**Keywords:** Hippocampus, Nucleus accumbens, Lateral hypothalamus, Prefrontal cortex, Viral tracing, ATLAS, Anterograde, Retrograde

## Abstract

The ventral hippocampus contributes to food intake regulation and a range of motivational and memory processes, and its dysfunction is associated with several cognitive and behavioral disorders. However, its circuit-level organization remains incompletely understood. Ventral CA1 (CA1v) neurons send projections to several regions involved in motivational control. Here we focus on three major forebrain targets: the nucleus accumbens shell (ACBsh), medial prefrontal cortex (mPFC), and lateral hypothalamic area (LHA). We mapped the upstream and downstream circuitry of CA1v neurons defined by their projections to these target regions in rats using complementary transsynaptic anterograde and retrograde viral tracing approaches. Monosynaptic outputs to ACBsh, mPFC, and LHA were targeted using ATLAS, a novel transsynaptic anterograde viral approach that drives Cre recombinase in neurons receiving synaptic transmission from the CA1v. Second-order projections arising from these defined pathways were then mapped using a Cre-dependent anterograde viral tracing strategy. In parallel, upstream inputs to CA1v neurons projecting to each downstream target were mapped using a conditional retrograde glycoprotein-deleted rabies viral approach. Anterograde tracing revealed both shared and pathway-specific second-order targets, including bidirectional CA1v projections. Retrograde tracing confirmed expected inputs (e.g., CA3) and uncovered previously unrecognized cortical sources that differed across downstream projection-defined CA1v subpopulations. Together, these findings delineate pathway-specific, multi-node circuits linking CA1v neurons to key motivational systems that may inform future therapeutic strategies for disorders involving ventral hippocampal dysfunction.

**Supplementary Information:**

The online version contains supplementary material available at https://doi.org/10.1007/s00429-026-03164-y.

## Introduction

The hippocampus (HPC), most widely known for its role in learning and memory processes, plays an important role in the etiology of many neuropsychiatric disorders, including depression (Bagot et al. 2015; Medrihan et al. 2022), schizophrenia (Mikell et al. 2009; Tseng et al. 2009), and substance abuse disorders (Rogers and See 2007; Bossert et al. 2016; Caban Rivera et al. 2023). The HPC also plays an important role in regulating reward-motivated behavior (Cooper et al. 2006; LeGates et al. 2018), influencing effort-based responding (Schmelzeis and Mittleman 1996), behavioral inhibition (Abela et al. 2013; Assari et al. 2025), and impulsivity (Abela et al. 2013; Hsu et al. 2018; Noble et al. 2019; Masuda et al. 2020). Recent and emerging work also reveals a critical role for the HPC in regulating appetite, caloric consumption, and body weight regulation (Kanoski and Grill 2017; Stevenson and Francis 2017; Barbosa et al. 2023), in part by modulating satiation and meal size control (Suarez et al. 2020), promoting cue-potentiated eating (Kanoski et al. 2013), and encoding meal-related memories (Hannapel et al. 2017, 2019; Décarie-Spain et al. 2022, 2025a; Yang et al. 2025). A better understanding of the neural circuitry via which the HPC contributes to the etiology of obesity, neuropsychiatric disorders, and disordered motivational outcomes could help to identify novel therapeutic targets.

While the hippocampus is often referred to a singular structure, this brain region contains several distinct subregions, each with their own distinguishable cytoarchitecture (Andersen 2007), genetic signature (Bienkowski et al. 2018), and connectivity (Kanoski and Grill 2017). In addition to the CA fields (1, 2, and 3) and the dentate gyrus (DG) subregional cellular distinctions that extend across the longitudinal axis, the rodent hippocampus is often divided into two larger subregions: the anterior “dorsal” hippocampus (dHPC) and the posterior “ventral” (vHPC), with some distinctions also including an “intermediate” HPC region (Fanselow and Dong 2010). The dHPC and vHPC subregions in the rodent are analogous to the posterior and anterior portions of the HPC in humans, respectively (Lee et al. 2019). The term ‘hippocampal formation’ refers to the hippocampus proper (CA and DG fields), entorhinal cortex (EC), and subiculum (SUB) (O’Keefe and Nadel 1978). Both the dorsal and ventral hippocampal regions have been shown to play a critical role in food intake and food-motivated behavior (Kanoski and Grill 2017; Hannapel et al. 2019), and these two subregions share common downstream targets—including the nucleus accumbens, subiculum, and septal nuclei (Fanselow and Dong 2010; Trouche et al. 2019; Barnstedt et al. 2024; Ibrahim et al. 2024). Despite these common downstream targets, these two hippocampal subregions are largely considered to be functionally and anatomically distinct (Fanselow and Dong 2010), with the vHPC established as being particularly important in food intake and food-motivated behaviors. More specifically, work from our group and others has established the ventral CA1 (CA1v) as an important regulator of food intake control, both with regards to cue-stimulated appetite, and satiation control (Hsu et al. 2015b, 2015a, 2018; Suarez et al. 2020; Décarie-Spain et al. 2025).

While CA1v is a critical node in the classic ‘tri-synaptic circuit’ of the hippocampus via its inputs from CA3v and via subiculum outputs (Andersen 2007), several important extra-hippocampal formation projections have been established. Our group and others have shown that the CA1v sends projections to the lateral septum (LS) (Sweeney and Yang 2015; Kosugi et al. 2021; Décarie-Spain et al. 2022), the medial prefrontal cortex (mPFC) (Chudasama et al. 2012; Hsu et al. 2018), the nucleus accumbens shell (ACBsh) (Bagot et al. 2015; Yang et al. 2020; Zhou et al. 2020; Tsai et al. 2022; Klug et al. 2025; Patterson et al. 2025), the basolateral amygdala (BLA) (Jimenez et al. 2018), and the lateral hypothalamic area (LHA) (Cenquizca and Swanson 2006; Hahn and Swanson 2010, 2012; Hsu et al. 2015c; Suarez et al. 2020; Décarie-Spain et al. 2025). Downstream projections from the CA1v to most of these targets have been shown to be relevant to food-motivated behavior and/or food consumption (Hsu et al. 2018; Décarie-Spain et al. 2022, Décarie-Spain et al. 2025), thus making them important potential targets for future obesity therapeutics.

**Fig. 1:**
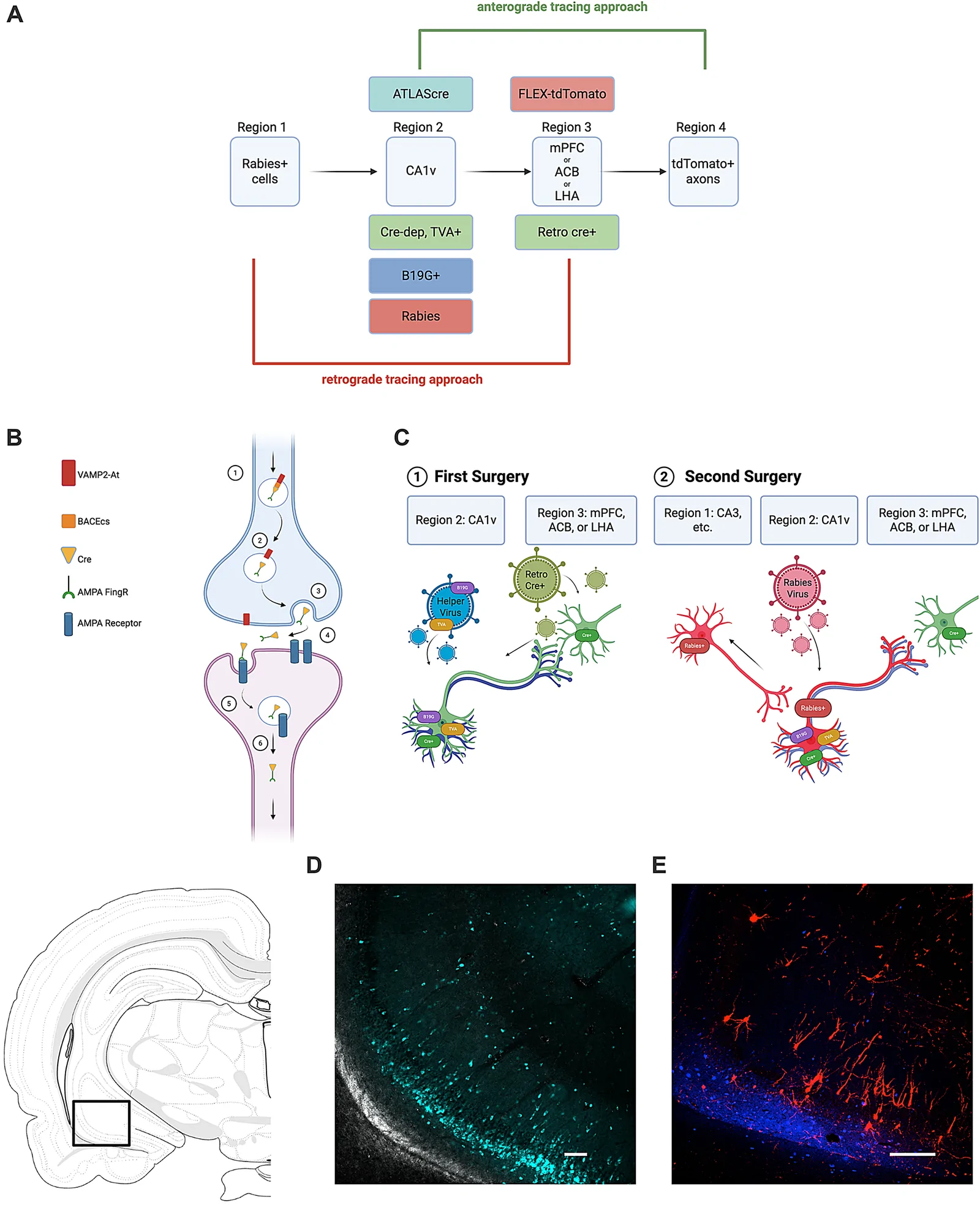
Methods for multi-node, multi-synaptic pathway tracing. **A** Schematic depicting both anterograde and retrograde multi-synaptic tracing approach. **B** Schematic depicting mechanism of action for ATLASsn_cre_ virus; (1) ATLASsn_cre_ is expressed in the presynaptic starter cell and targeted to the synapse via a VAMP2 domain. (2) A BACEcs is cleaved, (3) allowing AF-Cre to enter the synaptic cleft. (4) AF binds to GluA1 on the postsynaptic cell and (5) is endocytosed. (6) AF-cre moves to the nucleus of the postsynaptic cell, driving Cre expression (adapted from (Rivera et al. 2025). **C** Schematic depicting surgical approach for pseudo-rabies viral tracing approach; (1) in the first surgery, the rabies helper virus is infused into the CA1v driving expression of TVA receptor and rabies G-glycoprotein (B19G; necessary for transneuronal transfer of the rabies virus), along with a retro-cre inducing virus in 1 of 3 possible downstream regions. (2) A week later, in a second surgery, the rabies virus is infused into the CA1v, driving rabies expression in CA1v neurons which project to 1 of 3 possible downstream regions via EnvA expression (the ligand for the TVA receptor). **D** Representative image of ATLASsn_cre_ in the CAv (Scale bar, 100um). **E** Representative image of Rabies and Helper viruses in the CA1v (Scale bar, 500um)

Recent advances in viral-based neural tract tracing allow for transsynaptic-level insights, thus offering the potential to move beyond simple monosynaptic connections between regions to identify complex multi-node neuronal circuitry. To gain such insights for three distinct CA1v projection target pathways that are functionally linked with the control of food intake, here we use anterograde and retrograde transsynaptic viral tracing approaches to identify both downstream and upstream connections from and to, respectively, the CA1v-mPFC, CA1v-ACBsh, and CA1v-LHA projection pathways. The novel transsynaptic ATLAS approach (Rivera et al. 2025) provides us with the unique opportunity to probe this circuitry further by establishing what brain regions lie downstream of synaptic transmission from CA1v-mPFC, CA1v-ACBsh, and CA1v-LHA projections. This is in contrast to previous anterograde transsynaptic tracing approaches which may present synaptic leakage or retrograde transmission that are distinct from endogenous synaptic transmission (Zingg et al. 2017). Additionally, we utilized a transsynaptic glycoprotein-deleted retrograde rabies virus approach (Wall et al. 2010; Callaway and Luo 2015) to identify inputs into CA1v neurons based on their specific projection targets to mPFC, ACBsh, or LHA. Thus, in this paper we elucidate three distinct multi-node neuronal circuits centering on CA1v projections that are functionally relevant to food intake and body weight regulation (Fig. 1A).

## Methods and materials

### Animals

Male Sprague-Dawley rats (Envigo, Indianapolis, IN; postnatal day [PND] 60-70; 250-275g on avg upon arrival) were individually housed in a temperature-controlled vivarium with *ad libitum* access (except where noted) to water and food (LabDiet 5001, LabDiet, St. Louis, MO) on a 12h:12h reverse light/dark cycle. All procedures were approved by the Institute of Animal Care and Use Committee at the University of Southern California.

### Surgical procedures

For all surgical procedures, rats were anesthetized and sedated via intramuscular injections of ketamine (90 mg/kg), xylazine (2.8 mg/kg), and acepromazine (0.72 mg/kg). Animals were shaved, surgical site was prepped with iodine and ethanol swabs, and animals were placed in a stereotaxic apparatus for stereotaxic injections. Viruses were delivered using a microinfusion pump (Chemyx, Stafford, TX, USA) connected to a 33-gauge microsyringe injector attached to a PE20 catheter and Hamilton syringe. Flow rate was calibrated and set to 5 µl/min. Injectors were left in place for 2 min post injection, and animals were sutured following injections. Rats were also given analgesic (subcutaneous injection of ketoprofen [5mg/kg]) after surgery and once daily for 3 subsequent days thereafter. All rats recovered for at least three-weeks post-surgery prior to tissue collection to allow for viral transduction and expression. Anterograde and retrograde tracing surgeries took place in two different cohorts of animals (n=3 for each pathway).

### Viral injections

For neural tracing from second-order neurons receiving projections from the defined CA1v monosynaptic projection pathways, the following viruses were injected in one surgery: AAV8-ATLASsncre and AAV8-DIO-BACE-HA (ATLASsn_cre_; Gift from the Arnold Lab (Rivera et al. 2025); available on Addgene, ID 232351 and ID232353; 200nL per side) was unilaterally injected at the following coordinates: −4.9 mm anterior/posterior (AP), +-4.8 mm medial/lateral (ML), −7.8 mm dorsal/ventral (DV) (0 reference point at bregma for ML, AP, 0 reference point at skull surface near injection site for DV). Additionally, an AAV1-CAG-Flex-tdTomato-WPRE-bGH, (FLEX-tdTomato; UPenn Vector Core; 200nL per side) was unilaterally injected at one of the following coordinates, to allow for anterograde neural tracing from CA1v targets:

ACBsh: +1.2 mm AP, +-1.0 mm ML, −6.75 mm DV (0 reference point at bregma for ML, AP, 0 reference point at dura for DV).

LHA: -2.9 mm AP, +-1.0 mm and +- 1.6mm ML, −8.8 mm DV (0 reference point at bregma for all coordinates).

mPFC: +2.5 mm, AP, +-0.5 mm, −4.0 mm DV (0 reference point at bregma for all coordinates).

The ATLASsn_cre_ AAV drives expression of the AMPA.FingR (AF; FingR stands for fibronectin intrabody generated with mRNA display) attached to a synaptobrevin 2 (VAMP2) domain fused to a Synaptotagmin nanobody, thus targeting the ATLAS signaling molecule to the synapse. Additionally, the virus contains a beta secretase (BACE) cleavage site (BACEcs) between the AF and the VAMP2 domain, allowing endogenous BACE to cleave the AF from the VAMP2 domain and exit the lumen of the synaptic vesicle. Once in the synaptic cleft, AF, and its Cre payload, can bind to GluA1 on the postsynaptic cell, thereby being endocytosed and allowing for transmission of Cre in the postsynaptic cell.

For retrograde tracing from CA1v neurons based on their specific monosynaptic projection targets, surgeries were performed as previously described (Liu et al. 2017). The helper viruses AAV1-syn-FLEX-splitTVA-EGFP-tTA and AAV1-TREtight-mTagBFP2-B19G (Helper virus; Addgene, ID100798 and ID100799) were mixed 1:1 and unilaterally injected at the CA1v (600nL; see coordinates above), and a retrograde cre-expressing virus AAV-eSYN-EGFP-T2A-iCre-WPRE (Retro cre; Vector BioLabs, Cat #VB4855) was injected into either the ACB, LHA, or mPFC (400nL; see coordinates above). Seven days later, the rabies virus EnvA-SAD-B19-RVΔG tdTomato (Rabies; UC Irvine, Center for Neural Circuit Mapping, Cat. No: EnvA-RV-2, California, USA) was unilaterally injected into the CA1v (400nL). The helper virus, encoding for both a cre-dependent TVA receptor and the rabies G glycoprotein, were injected into the CA1v, along with a retro-cre virus injection in either the mPFC, ACBsh, or LHA. Rabies G is a necessary glycoprotein essential for transneuronal transfer of the rabies virus, while the TVA receptor is the receptor that the envelope protein from avian ASLV type A (EnvA) uses for cell entry. Importantly, TVA is not expressed in mammalian neurons, meaning that this combination of viral infusions ensures that the rabies G protein is expressed in all CA1v cells, while the TVA receptor expression is only driven in CA1v cells that project to one of our downstream regions of interest (ROI) (Fig. 1C). In a second surgery, a pseudotyped rabies virus is infused in the CA1v, one which lacks the Rabies G protein but expresses EnvA (Fig. 1C, E). Collectively, this surgical approach allows us to perform monosynaptic, retrograde tracing from CA1v neurons that project to either the ACBsh, LHA, or mPFC. Seven days later (14 days after the initial surgery), animals were euthanized, and brains were collected as described below.

For anterograde tracing controls, animals received an injection of AAV1-CAG-Flex-tdTomato-WPRE-bGH, (FLEX-tdTomato; UPenn Vector Core; 200nL per side) unilaterally in either the mPFC, ACBsh, or LHA, along with an injection of pENN.AAV.hSyn.TurboRFP.WPRE.RBG (TurboRFP; Addgene; 200nL per side) in the CA1v. For retrograde tracing controls; animals received a unilateral injection of EnvA-SAD-B19-RVΔG tdTomato (Rabies; 400nL) in the CA1v, along with an injection of AAV-eSYN-EGFP-T2A-iCre-WPRE (Retro cre; 400nL) in either the mPFC, ACBsh, or LHA. See above for all surgical coordinates.

### Immunohistochemistry (IHC) and histology

Rats were anesthetized and sedated with a ketamine (90 mg/kg)/xylazine (2.8 mg/kg)/acepromazine (0.72 mg/kg) cocktail, then transcardially perfused with 0.9% sterile saline (pH 7.4) followed by 4% paraformaldehyde (PFA) in 0.1 M borate buffer (pH 9.5; PFA). Brains were dissected out and post-fixed in PFA with 15% sucrose for 24 h, then flash frozen in isopentane cooled in dry ice. Full forebrains were sectioned to 30-µm thickness on a freezing microtome. Sections were collected in 5 series and stored in antifreeze solution at −20 °C until further processing. The hindbrain was not sectioned or analyzed.

Successful virally-mediated transduction was confirmed postmortem in all animals via IHC staining using immunofluorescence-based antibody amplification to enhance the fluorescence. Full forebrain IHC was then carried out in one representative animal using the following antibodies and dilutions: rabbit anti-RFP (1:2000, Rockland Inc., Limerick, PA, USA), and chicken anti-GFP (1:1000, Cat. no. ab13970, Abcam, Cambridge, UK), ALFA-TAG (1:1000, Cat No: N1502, NanoTag Biotechnologies, Gottingen, Germany). Antibodies were prepared in 0.02 M potassium phosphate-buffered saline (KPBS) solution containing 0.2% bovine serum albumin and 0.3% Triton X-100 at 4 °C overnight. After thorough washing with 0.02 M KPBS, sections were incubated in secondary antibody solution. All secondary antibodies were obtained from Jackson Immunoresearch and used at 1:500 dilution at 4 °C, with overnight incubations (Jackson Immunoresearch; West Grove, PA, USA). Sections were mounted and coverslipped using 50% glycerol in 0.02 M KPBS and the edges were sealed with clear nail polish. Photomicrographs of the entire forebrain and midbrain were acquired using either a Nikon 80i (Nikon DS-QI1,1280X1024 resolution, 1.45 megapixel) under epifluorescence or darkfield illumination. For all experiments, histological confirmation for inclusion in subsequent statistical analyses was based on identification of injections sites confined with the CA1v, mPFC, ACBsh, and LHA as observed using darkfield microscopy. Animals receiving the control surgeries (i.e. not given a cre-expressing virus) showed no cre-induced fluorescence (n=2-3/group; data not shown). The boundaries necessary for inclusion in studies for injection sites are depicted in Supplementary Figure 1A-D. Viral-labeled cell bodies were absent from dorsal subregions of the hippocampus.

### Axonal density and cell count quantification

Major sites of innervation for each transsynaptic pathway were confirmed in all animals (n=3 per pathway). Axonal labeling and cell counts from the forebrain of one representative animal per tracing technique was scored by a trained investigator and these scores were then entered into a custom built data-entry platform (Axiome C, created by JDH) built around Microsoft Excel software and designed to facilitate entry of data points for all gray matter regions across their atlas levels as described in a rat brain reference atlas: Brain Maps 4.0 (Swanson 2018). The Axiome C approach was used previously to facilitate the analysis of downstream projection targets in pathway tracing studies (Décarie-Spain et al. 2022; Hahn et al. 2022). For anterograde labeling, an ordinal scale, ranging from 0 (absent) to 7 (very strong) was used to record the weight of axonal density. For retrograde labeling, RFP+ cells were quantified using ImageJ (Rueden et al. 2017). All scoring was performed by a trained investigator. An average, total, and most common value was then obtained for each region across its atlas levels for which data were available. These data are summarized graphically for a representative animal on a brain flatmap summary diagram generated using a software-based approach (Hahn and Duckworth 2023; and partially adapted from (Décarie-Spain et al. 2022; Hahn and Duckworth 2023)), as well as on a bar graph with collapsing across some subregions (e.g., for LHA and LS) for the latter data depiction.

### Experimental design and statistical analyses

Data are represented as either modes, means, maximums, or total values. To provide the most representative assessment of axon density across regions of varying size and atlas coverage, we primarily emphasize the common value metric (mode axon density value across sections) in the main text, while additional analyses including total, average, and maximum axon density values are provided in the supplemental information.

## Results

### Anterograde tracing of second-order projections from three distinct brain regions receiving monosynaptic projections from the CA1v

In order to characterize downstream brain regions of the CA1v to mPFC, CA1v to ACBsh, and CA1v to LHA pathways, we employed a novel transsynaptic viral tracing approach, ATLAS, to label downstream neurons (and their projections) in a strictly anterograde manner mediated by endogenous synaptic transmission of Cre in the postsynaptic cell (Rivera et al. 2025) (Fig. 1B). We unilaterally injected ATLASsn_cre_ in the CA1v (Fig. 1D) and FLEX-tdTomato in either the mPFC, ACBsh, or LHA to trace downstream targets of these pathways (Fig. 1A).

**Fig. 2:**
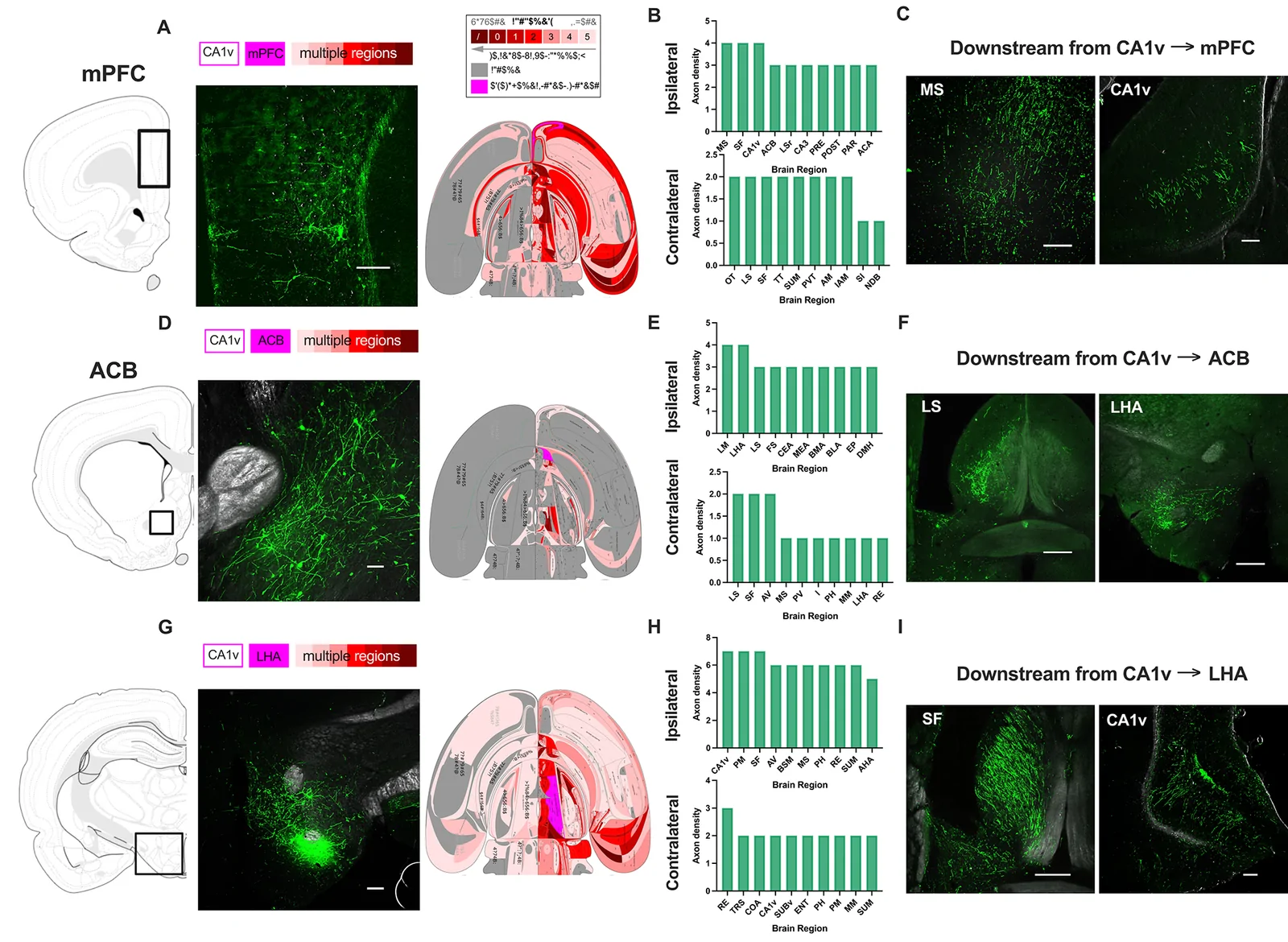
Most common axon density in anterograde tracing of 2^nd^-order projections from CA1v pathways. **A** Representative image (left) and flatmap (right) depicting most common axon density for CA1v-mPFC anterograde tracing. **B** Ipsilateral (top) and contralateral (bottom) graphs depicting most common axon density rating in CA1v-mPFC pathway. **C** Representative images depicting axon density in the RE (left) and CA1v (right). **D** Representative image (left) and flatmap (right) depicting most common axon density for CA1v-ACB anterograde tracing. **E** Ipsilateral (top) and contralateral (bottom) graphs depicting most common axon density rating in CA1v-ACB pathway. **F** Representative images depicting axon density in the LS (left) and LHA (right).** G** Representative image (left) and flatmap (right) depicting most common axon density for CA1v-LHA anterograde tracing. **H** Ipsilateral (top) and contralateral (bottom) graphs depicting most common axon density rating in CA1v-LHA pathway. **I** Representative images depicting axon density in the FS (left) and CA1v (right)

Forebrain- and midbrain-wide neuroanatomical imaging and quantification of axon density revealed that mPFC neurons receiving projections from the CA1v send dense projections throughout the brain, with the medial septum (MS) and CA1v having among the highest most common value in terms of axon density (Fig. 2A-C), while the CA1v had high average axon density (Supp. Figure 2A,B), and the lateral septum (LS), lateral hypothalamic area (LHA) , and the anterior cingulate area (ACA) showed among the greatest total axon density (Supp. Figure 3A,B) on the side ipsilateral to the injection site. On the contralateral side, the LS and olfactory tubercle (OT) had among the highest mode for axon density, while the septofimbrial nucleus (SF) and anteromedial thalamic nucleus (AM), and LS and paraventricular thalamic nucleus (PVT), scored the highest in terms of average and total axon density respectively (Supp. Figure 2A,B; Supp. Figure 3A,B). When analyzing for the maximum axon density, the CA1v and LS scored among the highest on both the ipsilateral and contralateral side of the CA1v-mPFC pathway (Supp. Figure 4A,B). For a complete list of brain regions with axons from the CA1v-mPFC pathway, see Supplemental tables 1-4.

The brain regions with among the highest mode of axon density that are innervated by the Ca1v-ACBsh pathway include the LS and the LHA (Fig. 2D-F), while the substantia innominata (SI), lateral mamillary nucleus (LM), and mediodorsal thalamic nucleus medial part (MDm) had among the greatest average axon density on the ipsilateral side (Supp. Figure 2C,D). When examining projections contralateral to the injection site, the LS and the SF were among the highest scoring regions for both mode and average of axon density (Fig. 2E; Supp. Figure 2C, D) Finally, the LHA, bed nuclei of terminal stria (BST), and the LS had particularly high total axon density in the CA1v-ACBsh pathway on the ipsilateral side, and the LS and PVT had among the highest total axon density on the contralateral side (Supp. Figure 3C,D). The LS also had strong maximum axon density score on both the ipsilateral and contralateral sides (Supp. Figure 4C, D). For a complete list of brain regions with axons from the CA1v-ACBsh pathway, please see Supplemental tables 1-4.

LHA neurons receiving projections from the CA1v project to multiple downstream brain regions, with the septofimbrial nucleus (SF) and CA1v being among the most robust downstream targets when analyzing by most common axon density value (Fig. 2G-I) and by average axon density value on the ipsilateral side (Supp. Figure 2E,F). On the side of the brain contralateral to tracing injections, the RE and triangular septal nucleus (TRS) had among the highest mode for axon density, while the RE and medial mammillary nucleus (MM) had the highest average axon density (Fig. 2H; Supp Figure 2E,F).When analyzing for total axon density per region the BST, LS, and periaqueductal gray (PAG) scored among the highest on the ipsilateral side, and the LS and PAG scored among the highest in terms of contralateral projections, though the contralateral LHA had the highest total axon density (Supp. Figure 3E,F). Finally, when analyzing for maximum axon density in second-order projections, the CA1v and dorsomedial hypothalamus (DMH) scored among highest on the ipsilateral side, while the mammillary nucleus (MM) and the medial septum (MS) scored among highest on the contralateral side (Supp. Figure 4E, F). For a complete list of brain regions with axons from the CA1v-LHA pathway, see Supplemental tables 1-4.

### Retrograde tracing from CA1v neurons that project to either the ACBsh, LHA, or mPFC

To characterize the upstream brain regions of the CA1v-ACBsh, CA1v-LHA, and CA1v-mPFC pathways, we utilized a monosynaptic retrograde rabies viral approach, allowing us to perform monosynaptic and retrograde tracing solely from CA1v neurons which further project to either the mPFC, ACB, or LHA (Fig. 1A).

**Fig. 3:**
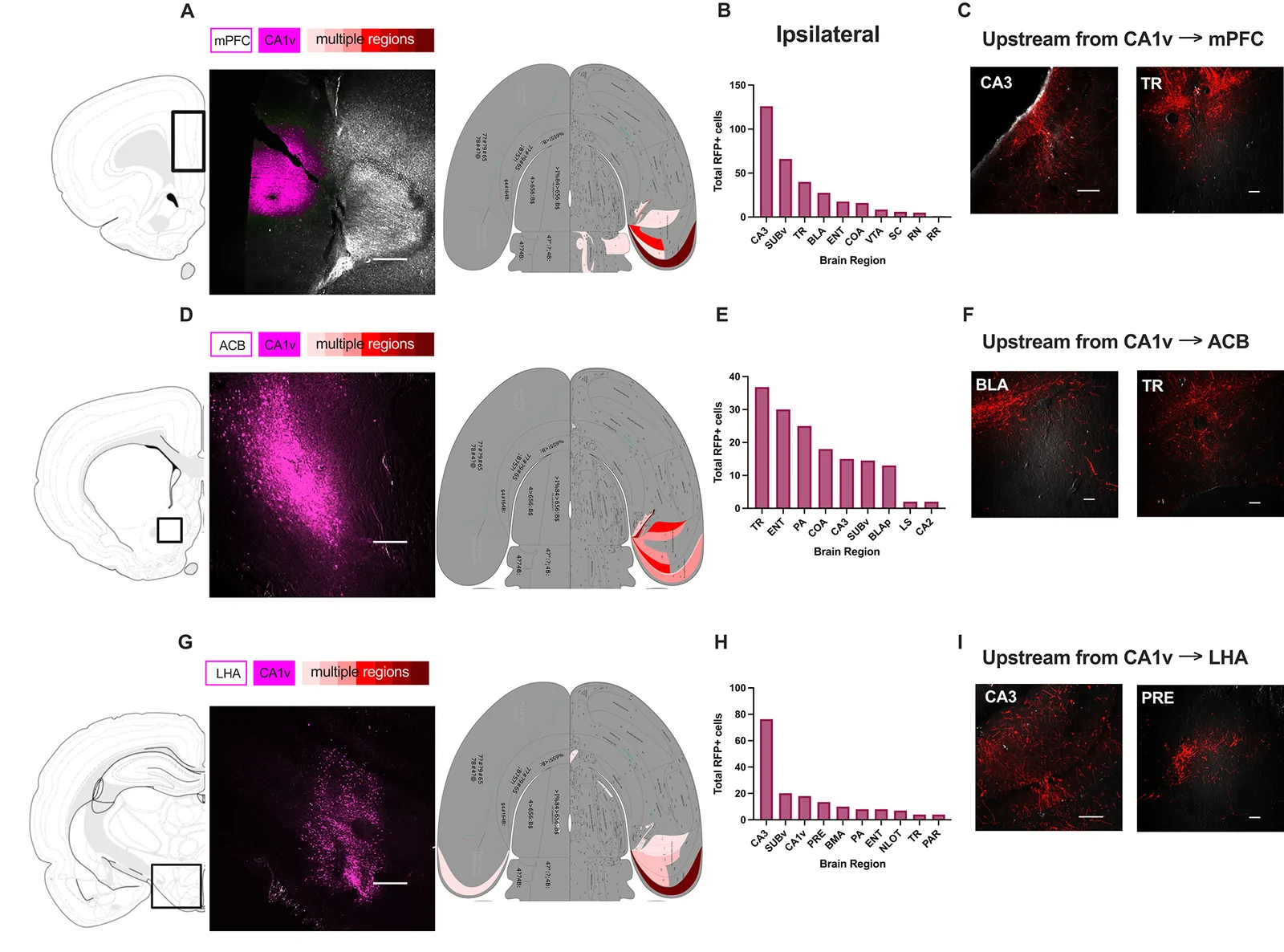
Total RFP+ cells in retrograde tracing of primary inputs to CA1v pathways. **A** Representative image of AAVretro-cre injection site (left; pink) and flatmap (right) depicting total RFP+ cell bodies for CA1v-mPFC retrograde tracing. **B** Ipsilateral graph depicting total RFP+ cells for retrograde tracing in CA1v-mPFC pathway. **C** Representative images depicting cell bodies in the TR (left)and CA3v (right). **D** Representative image of AAVretro-cre injection site (left; pink) and flatmap (right) depicting total RFP+ cell bodies for CA1v-ACB retrograde tracing. **E** Ipsilateral graph depicting total RFP+ cells for retrograde tracing in CA1v-ACB pathway. **F** Representative images depicting cell bodies in the BLA (left) and TR (right)**G** Representative image of AAVretro-cre injection site (left; pink) and flatmap (right) depicting total RFP+ cell bodies for CA1v-LHA retrograde tracing. **H** Ipsilateral graph depicting total RFP+ cells for retrograde tracing in CA1v-LHA pathway. **I** Representative images depicting cell bodies in the CA3v (left) and PRE (right)

Our results reveal that the CA3 and the ventral subiculum (SUBv) give robust input to both the CA1v-mPFC and CA1v-LHA pathways (Fig. 3A-C, G-I; Supp. Figure 5 A,B,E,F). While the CA1v-ACBsh also receives input from the CA3, a majority of inputs to this pathway are cortical, including brain regions such as the posterior amygdalar nucleus (PA) and the cortical nucleus of the amydgala (COA) (Fig. 3D-F; Supp. Figure 5C,D). Additionally, the postpiriform transition area (TR) and the basolateral amygdala (BLA) are among the top projections to both the CA1v-mPFC and CA1v-ACBsh pathways, while each projects minimally to the CA1v-LHA pathway (Fig. 3A-F, Supp. Figure 5). Of note, the VTA also sends projections to the CA1v-mPFC pathway, albeit minimally (Fig. 3A,B; Supp. Figure 5B).

## Discussion

**Fig. 4:**
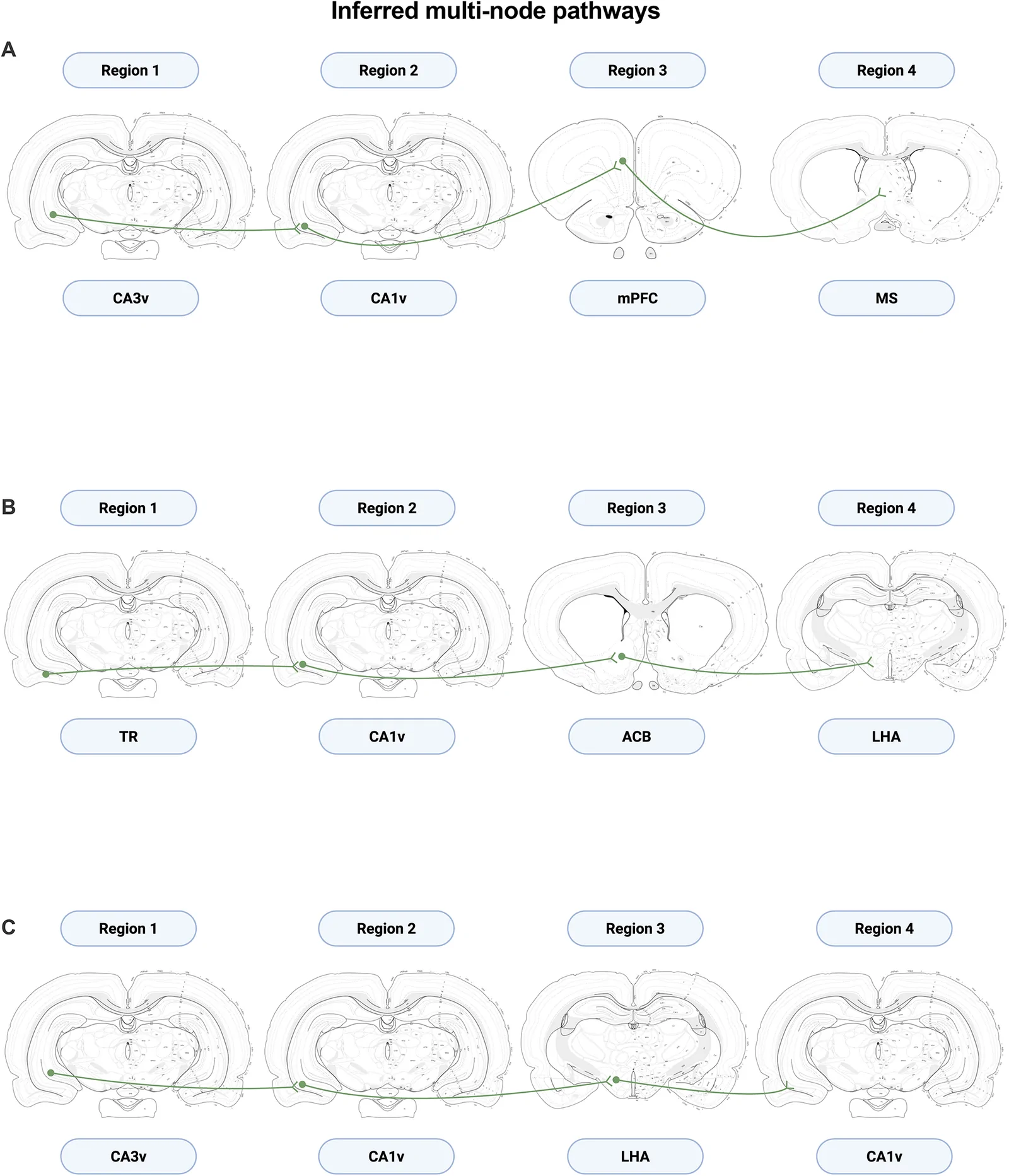
Inferred multi-node pathways. **A** Inferred multi-node pathway centered around CA1v-mPFC projections. **B** Inferred multi-node pathway centered around CA1v-ACBsh projections. **C** Inferred multi-node pathway centered around CA1v-LHA projections

A better understanding of CA1v pathways regulating motivation for food and other reinforcers could facilitate the development of novel therapeutics for the treatment of obesity and neuropsychiatric disorders. Recent advances in transsynaptic anterograde tracing technology (Rivera et al. 2025), paired with established transsynaptic retrograde tracing approaches, have allowed us to uncover multi-node neuronal circuits connecting the CA1v to brain regions crucial for reward, emotional, and contextual processing. Our results in male Sprague Dawley rats outline three distinct, 4-node, inferred multi-synaptic pathways anchored from known monosynaptic CA1v projection pathways previously implicated in motivational control (Fig. 4). While experiments were only carried out in males, rodent neural tract tracing data to date do not reveal sex differences in projections from these brain regions (Swanson et al. 2025). Using a unique combination of tracing approaches, we reveal both upstream and second-order downstream targets of the CA1v-mPFC, CA1v-ACBsh, and CA1v-LHA projection pathways. It has been well established that the CA1v-mPFC, CA1v-ACB, and CA1v-LHA (Décarie-Spain et al. 2025) projections play a role in hippocampal-dependent memory, but there is also a clear role for these pathways in both food intake and food-motivated behaviors (Chudasama et al. 2012; Hsu et al. 2015b, 2018; Trouche et al. 2019; Suarez et al. 2020; Klug et al. 2025). These results confirm previous work showing that projections from this pathway are primarily ipsilateral (Lee et al. 2024), with contralateral targets largely mirroring the ipsilateral projection pattern for each pathway, with some exceptions for the CA1v-mPFC pathway.

One notable outcome of this study is that there are several common downstream brain regions receiving projections from these distinct CA1v-derived pathways. For example, the LS, known for its role in motivation and emotion (Wirtshafter and Wilson 2021), is not only a monosynaptic target of CA1v neurons (Décarie-Spain et al. 2022), but also emerged as a common robust second-order target of both the CA1v-mPFC and CA1v-ACBsh pathways. In this capacity, the LS may receive integrated multi-modal information from direct and indirect HPC projections to mediate goal-directed behavior. Additionally, the LS is increasingly being recognized for its role in eating-related behavior (Sweeney and Yang 2015; Kosugi et al. 2021), and previous research identified a role for melanin-concentrating hormone (MCH) signaling in the LS in promoting food intake (Payant et al. 2025). Given that MCH-expressing neurons in the LHA that project to the CA1v have also been implicated in food-motivated behavior (Noble et al. 2019), these results suggest that the LS may play a central integrating role for energy balance-relevant information conveyed by hippocampal-originating cortical and striatal pathways, as well as directly from hypothalamic MCH signaling.

Another shared downstream target of these pathways is the BST. Both the CA1v-ACBsh and CA1v-LHA pathways send robust 2^nd^-order projections to the BST, a brain region heavily implicated in the integration of motivational, affective, and stress-related signals (Wang et al. 2025) (details of BST subregions receiving projections are given in Supplemental Tables 1-4). Interestingly, the BST has been increasingly implicated in food intake control (Wang et al. 2025), particularly reward-motivated behavior (Ge and Balleine 2022; Huijgens et al. 2024), such as impulsive operant responding (Kim et al. 2018). As a 2^nd^-order target site in these pathways, the BST may serve as a point of convergence for hippocampal outputs related to motivational context and internal state**.** The confluence of CA1v-derived neural activity on the BST may influence downstream circuits involved in control of energy balance and reward processing.

Previous work describing the connectivity of CA1v neurons suggests that the majority are single target projection neurons (Gergues et al. 2020). For example, only ~25% of CA1v neurons project to two or more downstream targets, with only ~3% of PFC projecting neurons also projecting to the LHA, ~6% of PFC neurons also projecting to the ACB, and ~4% of ACB neurons also projecting to the LHA. Although the current results indicate relatively few CA1v neurons project to more than one region, the shared 2^nd^-order projections between CA1v-derived pathways may allow functionally distinct hippocampal outputs to converge and be integrated downstream. Gergues et al. (2020) also found that CA1v neurons which project to more than one brain region do so in a non-random fashion, with CA1v neurons which project to both the LS and the mPFC or ACB being more common than would be expected if projections were bionomially distributed. The role of the LS as a shared 2^nd^-order projection of CA1v-mPFC and CA1v-ACBsh pathways suggests that hippocampal input to the LS is distributed across multiple output streams and may represent an important downstream node within hippocampal circuits relevant to reward-motivated behavior. Further, mPFC-projecting CA1v neurons have a unique transcriptional profile relative to subcortically projecting CA1v neurons specifically enriched for genes involved in metabolic processes (Gergues et al. 2020). This suggests that the CA1v-mPFC may be positioned to integrate metabolic signals with cognitive processes.

The CA1v itself is a common 2^nd^-order projection target in both the CA1v-mPFC and CA1v-LHA pathways, thus providing evidence of a putative recurrent circuit connection. Furthermore, we know from previous studies that the CA1v neurons which project to the ACBsh themselves receive projections from the LHA (Noble et al. 2019), and results above show that the CA1v-ACBsh pathway sends 2^nd^-order projections to the LHA as well. Thus, the CA1v is a shared downstream region of interest in all three CA1v-derived pathways. Previous research on recurrent circuits have identified feedback loops based on structure alone. Advancing these findings, the novel ATLAS approach allows us to identify recurrent CA1v circuitry based on physiological synaptic communication. To our knowledge, this is the first study identifying definitive recurrent circuit neurons driven by empirical results vs. inference made from common/shared known connections. This recurrent connectivity suggests the presence of feedback loops from and to the vHPC, which may allow hippocampal activity to be dynamically modulated by downstream striatal, cortical, and hypothalamic inputs. While we do not know which specific neurons in the CA1v these pathways project onto, it is possible that inhibitory interneurons (IIs) within the CA1v participate in these feedback loops, allowing for fine tuning of glutamatergic hippocampal output via intra-hippocampal inhibitory feedback. IIs are known to play a crucial role in regulating circuit dynamics in the HPC for memory, fine tuning both the firing timing and frequency of primary cells in the HPC (Topolnik and Tamboli 2022). It is possible that 2^nd^-order downstream projections recurrently connect back to the CA1v to inhibitory interneurons to fine tune hippocampal output based on motivational or homeostatic state.

Pathway-specific transsynaptic retrograde tracing revealed significant input to the CA1v from the intrinsic hippocampal regions, with the CA3v not surprisingly being a primary source of input to CA1v in both the CA1v-mPFC and CA1v-LHA pathways. However, while the CA1v-ACBsh pathway also received some synaptic input from the CA3v, a larger portion of its inputs were cortical, coming from the posterior amygdalar nucleus (PA), cortical nucleus of the amygdala (COA), and the basolateral amygdalar nucleus (BLA). Previous research has shown a role for amygdalar inputs to the ACB in regulating reward motivated behavior (Millan et al. 2017; Poggi et al. 2024; Taniguchi et al. 2026), and one study found that long-term potentiation (LTP) at the vHPC-ACB synapse was contingent to BLA-ACB activation (Yu et al. 2022). In this case, it was concluded that vHPC inputs to the ACB acquire increase circuit representation via the contingent BLA-ACB activation. While our identified pathway is not a direct amygdala to ACB circuit, perhaps these amygdalar inputs to the CA1v-ACBsh pathway integrate emotion and motivational context with recurrent hippocampal output pathways to influence accumbal processing. Additionally, the presence of midbrain inputs, such as the VTA in the CA1v-mPFC pathway, suggests that internal state signals related to motivation may modulate hippocampal-prefrontal communication.

There are limitations in the methodology of the current study worth noting. First, while dense axonal labelling likely indicates some terminal field presence, our approach did not resolve terminals from axons of passage for our anterograde tracing approach (e.g., via co-labeling of presynaptic terminal markers). Second, for the transsynaptic retrograde tracing, the current viral technology does not allow us to distinguish targeted neurons within the CA1v, leaving open the possibility that there are intra-CA1v projections—i.e. distinct CA1v neurons which give input to our primary CA1v neurons—in these pathways that we cannot identify from this approach. Additionally, unlike ATLAS, neither the rabies virus nor the retro-cre-inducing virus used in our retrograde tracing approach are mediated by endogenous synaptic transmission.

Another limitation of the present study is that our analyses were restricted to forebrain structures, and therefore potential hindbrain targets and broader brainstem connectivity of these pathways were not assessed. Finally, while CA1v projections neurons are presumed to be glutamatergic, previous studies have identified a small population of GABAergic projection neurons within the CA1v (Melzer et al. 2012), thus potentially limiting the specificity of the pathway mapping with regards to hippocampal neural neurotransmitter profile. However, there is currently no evidence that these inhibitory project neurons project to the medial prefrontal cortex, nucleus accumbens shell, or lateral hypothalamic area, and thus are anterograde projection results are likely targeting glutamatergic synapses exclusively.

In summary, our results reveal downstream and upstream neuronal projections from three distinct, multi-node pathways anchored in the CA1v, thus providing a framework for inferred 4-node neural pathways (Fig. 4). The CA1v-mPFC, CA1v-ACBsh, and CA1v-LHA pathways share common upstream targets, namely the CA3v and SUBv, though the CA1v-ACBsh pathway receives substantial innervation from cortical areas. Additionally, these projection pathways share common downstream targets, namely the LS for the CA1v-mPFC and CA1v-ACBsh projections, and the BST for the CA1v-ACBsh and CA1v-LHA projections. These common downstream targets suggest that anatomically distinct CA1v-centered pathways converge onto shared downstream nodes, potentially enabling distributed yet coordinated influence over downstream subcortical circuits involved in motivation and internal state regulation. Finally, the CA1v itself is a shared downstream projection site of the CA1v-mPFC and CA1v-LHA pathways, and such recurrent/bidirectional connectivity suggests the existence of vHPC recurrent circuits that allow CA1v activity to be reciprocally and dynamically modulated by downstream inputs. Future research should investigate how environmental exposures might affect the connections and strength of connections within these pathways, particularly exposure to a Western diet (WD). Previous research suggests that early development, and the hippocampus specifically, is particularly sensitive to WD exposure (Tsan et al. 2022; Hayes et al. 2024b, 2024a) and future research should examine if and how early-life WD exposure effects development of these motivational control-relevant connectivity circuits. Additionally, future studies should aim to genetically phenotype first-order projection neuron targets in these pathways, allowing us to better understand the nature of the recurrent connectivity discovered in some of these pathways.

## Supplementary Information

Below is the link to the electronic supplementary material.

Supplementary file1 (PDF 740 KB) Supplemental Figure 1. Inclusion criteria for histology. Swanson Atlas images depicting boundaries for inclusion in study for the A. mPFC B. ACB C. CA1v and D. LHA

Supplementary file2 (PDF 1219 KB) Supplemental Figure 2. Average axon density for anterograde tracing of 2nd order projections from CA1v pathways. A. Flatmap depicting average axon density for CA1v-mPFC anterograde tracing. B. Ipsilateral (left) and contralateral (right) graphs depicting average axon density rating in CA1v-mPFC pathway. C. Flatmap depicting average axon density for CA1v-ACB anterograde tracing. D. Ipsilateral (left) and contralateral (right) graphs depicting average axon density rating in CA1v-ACB pathway. E. Flatmap depicting average axon density for CA1v-LHA anterograde tracing. F. Ipsilateral (left) and contralateral (right) graphs depicting average axon density rating in CA1v-LHA pathway

Supplementary file3 (PDF 1230 KB) Supplemental Figure 3. Total axon density for anterograde tracing of 2nd order projections from CA1v pathways. A. Flatmap depicting total axon density for CA1v-mPFC anterograde tracing. B. Ipsilateral (left) and contralateral (right) graphs depicting total axon density rating in CA1v-mPFC pathway. C. Flatmap depicting total axon density for CA1v-ACB anterograde tracing. D. Ipsilateral (left) and contralateral (right) graphs depicting total axon density rating in CA1v-ACB pathway. E. Flatmap depicting total axon density for CA1v-LHA anterograde tracing. B. Ipsilateral (left) and contralateral (right) graphs depicting total axon density rating in CA1v-LHA pathway

Supplementary file4 (PDF 1227 KB) Supplemental Figure 4. Maximum axon density for anterograde tracing of 2nd-order projections from CA1v pathways. A. Flatmap depicting maximum axon density for CA1v-mPFC anterograde tracing. B. Ipsilateral (left) and contralateral (right) graphs depicting maximum axon density rating in CA1v-mPFC pathway. C. Flatmap depicting maximum axon density for CA1v-ACB anterograde tracing. D. Ipsilateral (left) and contralateral (right) graphs depicting maximum axon density rating in CA1v-ACB pathway. E. Flatmap depicting maximum axon density for CA1v-LHA anterograde tracing. B. Ipsilateral (left) and contralateral (right) graphs depicting maximum axon density rating in CA1v-LHA pathway

Supplementary file5 (PDF 1243 KB) Supplemental Figure 5. Average RFP+ cell bodies in retrograde tracing of primary inputs to CA1v pathways. A. Flatmap depicting average RFP+ cell bodies for CA1v-mPFC retrograde tracing. B. Ipsilateral graph depicting average RFP+ cells for retrograde tracing in CA1v-mPFC pathway. C. Flatmap depicting average RFP+ cell bodies for CA1v-ACB retroograde tracing. D. Ipsilateral graph depicting average RFP+ cells for retrograde tracing in CA1v-ACB pathway. E. Flatmap depicting average RFP+ cell bodies for CA1v-LHA retrograde tracing. F. Ipsilateral graph depicting average RFP+ cells for retrograde tracing in CA1v-LHA pathway. G. Contralateral graph depicting total RFP+ cells for retrograde tracing in CA1v-LHA pathway. H. Contralateral graph depicting total RFP+ cells for retrograde tracing in CA1v-LHA pathway

Supplementary file6 (XLSX 78 KB) Supplemental Table 1. All most common axon density ratings for anterograde tracing of 2nd-order projections from CA1v pathway. Supplemental Table 2. All average axon density ratings for anterograde tracing of 2nd-order projections from CA1v pathways. Supplemental Table 3. All total axon density ratings for anterograde tracing of 2nd-order projections from CA1v pathways. Supplemental Table 4. All maximum axon density ratings for anterograde tracing of 2nd-order projections from CA1v pathways.

## Data Availability

The datasets generated and analyzed in the current study are available upon request.

## Abbreviations

ACA: Anterior cingulate area
ACB: Nucleus accumbens
AD: Anterodorsal nucleus thalamus
AHN: Anterior hypothalamic nucles
AM: Anteromedial nucleus thalamus
AV: Anteroventral nucleus thalamus
BLA: Basolateral nucleus amygdala
BMA: Basomedial nucleus amygdala
BSM: Bed nucleus of the stria medullaris
BST: Bed nuclei stria terminalis
CA1d: Dorsal field CA1, Ammon’s horn
CA1v: Ventral field CA1, Ammon’s horn
CA2: Field CA2, Ammon’s horn
CA3: Field CA3, Ammon’s horn
CLA: Claustrum
COA: Cortical nucleus amygdala
COM: Commisural nucleus of the stria terminalis
CP: Caudoputamen
DMH: Dorsomedial nucleus hypothalamus
ENT: Entorhinal area
EP: Endopiriform nucleus
I: Internuclear hypothalamic area
IAM: Interanteromedial nucleus thalamus
LHA: Lateral hypothalamic area
LM: Lateral mammillary nucleus
LS: Lateral septum
MD: Mediodorsal nucleus thalamus
MEA: Medial nucleus amygdala
MM: Medial mammillary nucleus
MO: Motor areas
MRN: Mesencephalic reticular nucleus
MS: Medial septal nucleus
NLOT: Nucleus of the lateral olfactory tract
OT: Olfactory tubercle
PA: Posterior nucleus amgydala
PAA: Piriform-amygdalar area
PAG: Periaqueductal gray
PAR: Parasubiculum
PD: Posterodorsal preoptic nucleus
PH: Posterior hypothalamic nucleus
PM: Premammillary nucleus
POST: Postsubiculum
PRE: Preoptic region
PT: Parataenial nucleus
PV: Periventricular nucleus hypothalamus
PVH: Paraventricular nucleus hypothalamus
PVT: Paraventricular nucleus thalamus
RE: Nucleus reuniens
RN: Red nucleus
RR: Mesencephalic reticular nucleus
RSP: Retrosplenial area
SC: Superior colliculus
SF: Septofimbrial nucleus
SI: Substantia innominata
SUBd: Dorsal subiculum
SUBv: Ventral subiculum
SUM: Supramammillary nucleus
TM: Tuberomammillary nucleus
TR: Postpiriform transition area
TT: Tenia tecta
TU: Tuberal nucleus
VTA: Ventral tegmental area
ZI: Zona incerta
