# Supplementary figures and images for: Transsynaptic neural circuit mapping of ventral hippocampus motivational control systems
Source: Brain Struct Funct. 2026 Jul 22;231(7):106. doi: 10.1007/s00429-026-03164-y (PMC13391668; doi:10.1007/s00429-026-03164-y)

# Supplemental Figure 1.

**A**

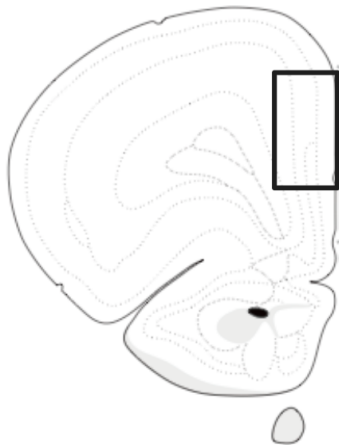

**B**

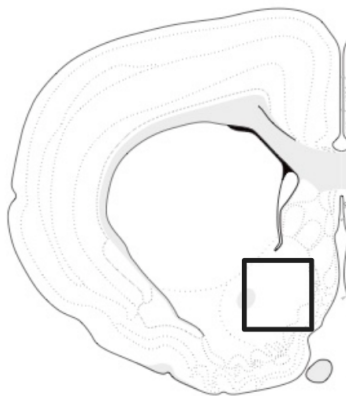

**C**

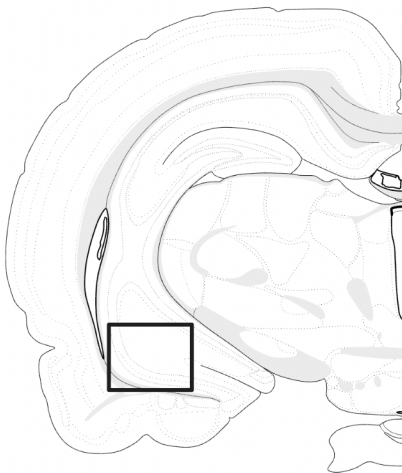

**D**

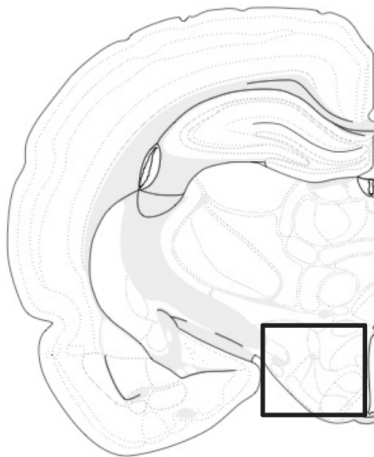

Supplement: Supplementary file 1 — Supplementary file1 (PDF 740 KB) Supplemental Figure 1. Inclusion criteria for histology. Swanson Atlas images depicting boundaries for inclusion in study for the A. mPFC B. ACB C. CA1v and D. LHA [file 429_2026_3164_MOESM1_ESM.pdf]

Supplemental Figure 2.

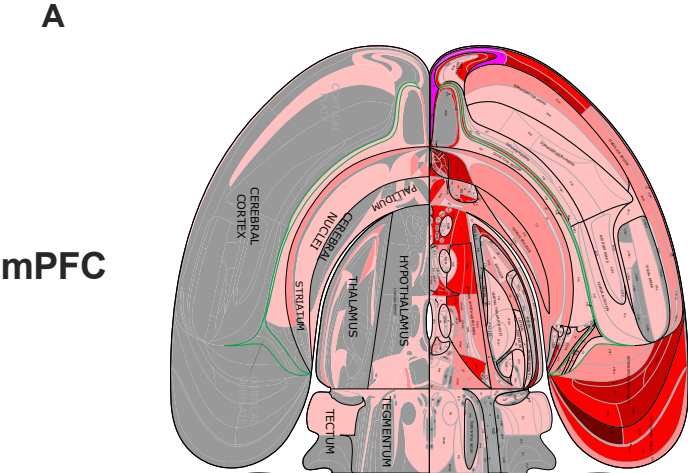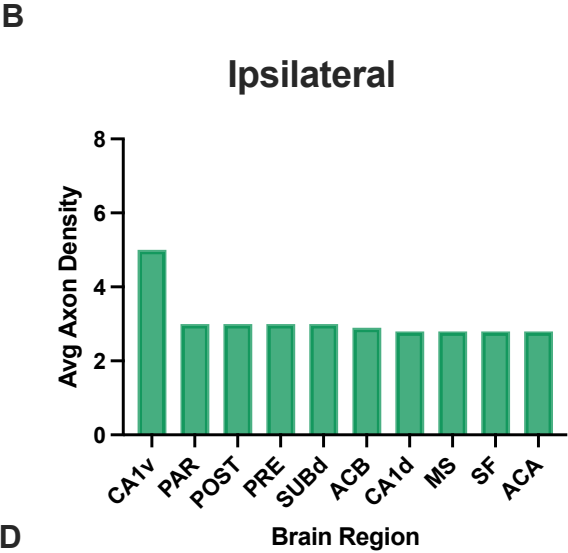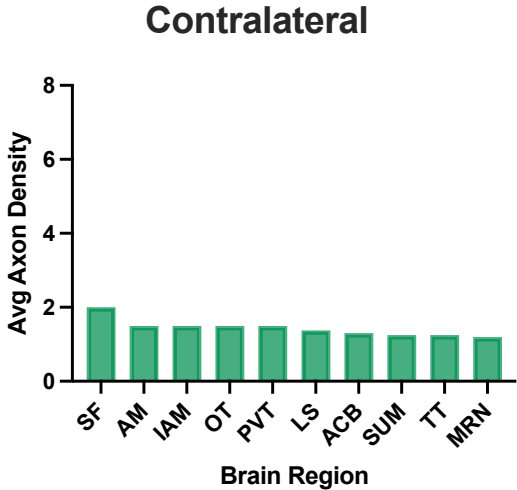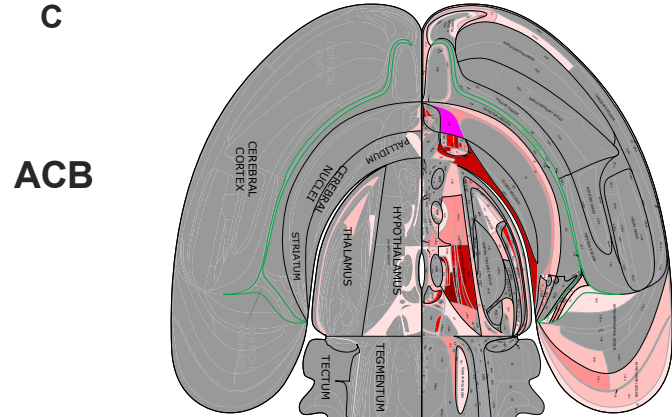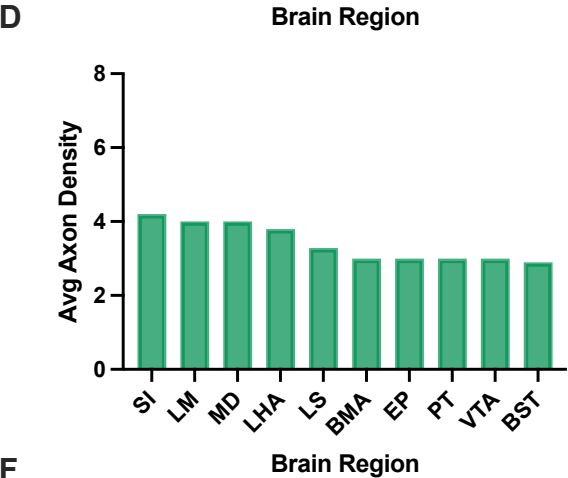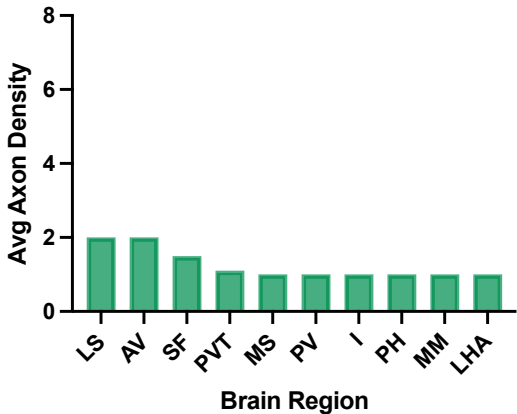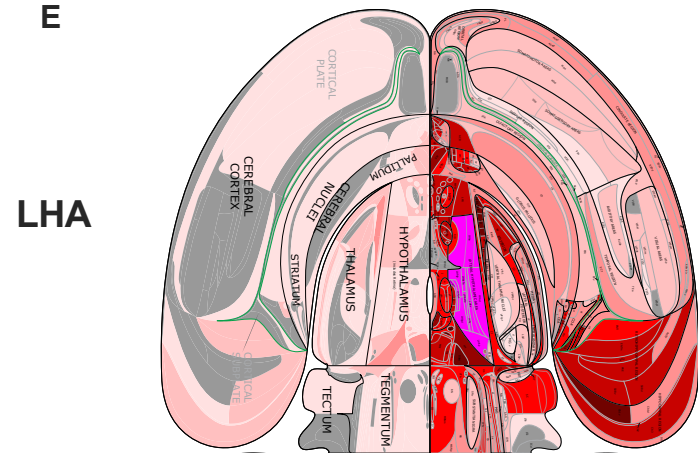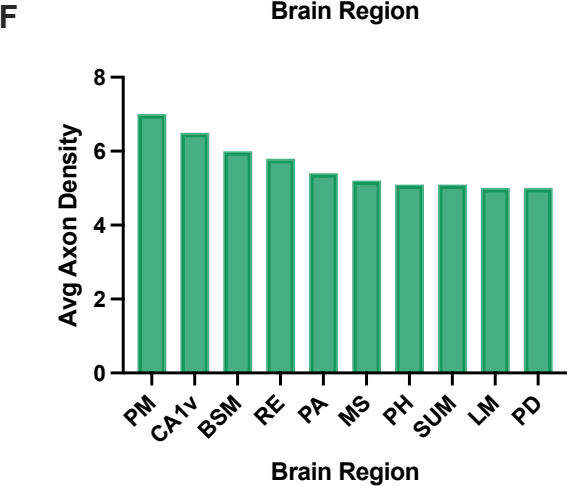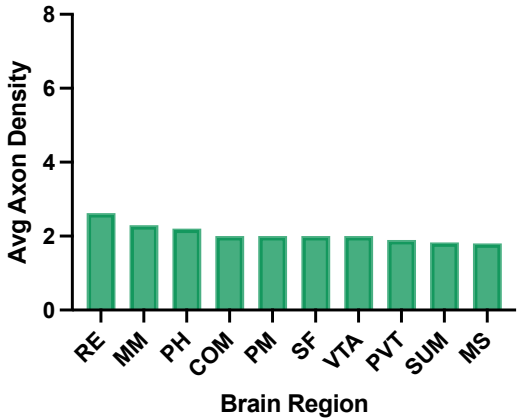

Supplement: Supplementary file 2 — Supplementary file2 (PDF 1219 KB) Supplemental Figure 2. Average axon density for anterograde tracing of 2nd order projections from CA1v pathways. A. Flatmap depicting average axon density for CA1v-mPFC anterograde tracing. B. Ipsilateral (left) and contralateral (right) graphs depicting average axon density rating in CA1v-mPFC pathway. C. Flatmap depicting average axon density for CA1v-ACB anterograde tracing. D. Ipsilateral (left) and contralateral (right) graphs depicting average axon density rating in CA1v-ACB pathway. E. Flatmap depicting average axon density for CA1v-LHA anterograde tracing. F. Ipsilateral (left) and contralateral (right) graphs depicting average axon density rating in CA1v-LHA pathway [file 429_2026_3164_MOESM2_ESM.pdf]

Supplemental Figure 3.

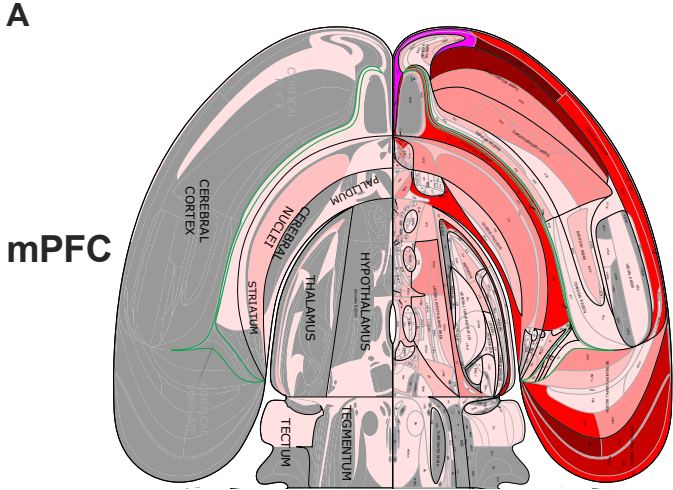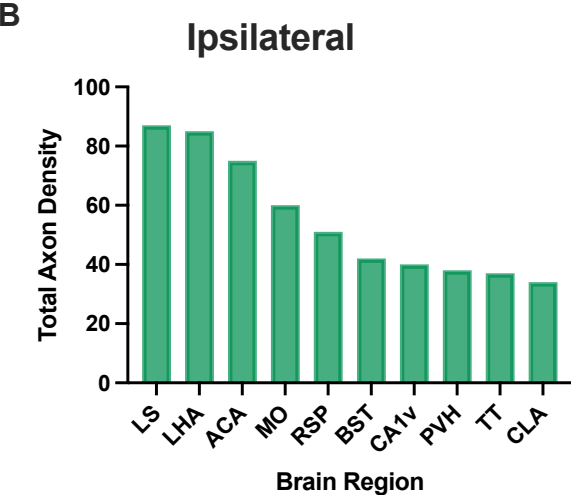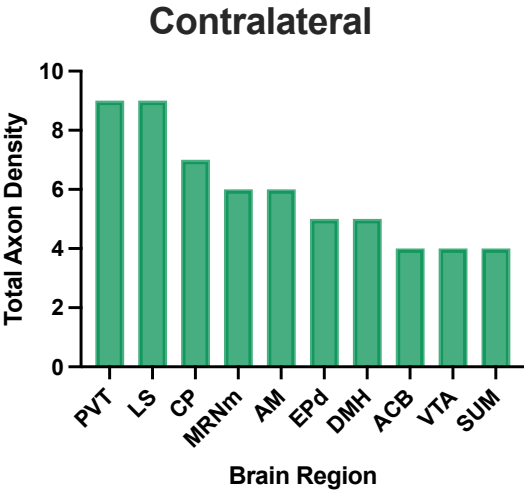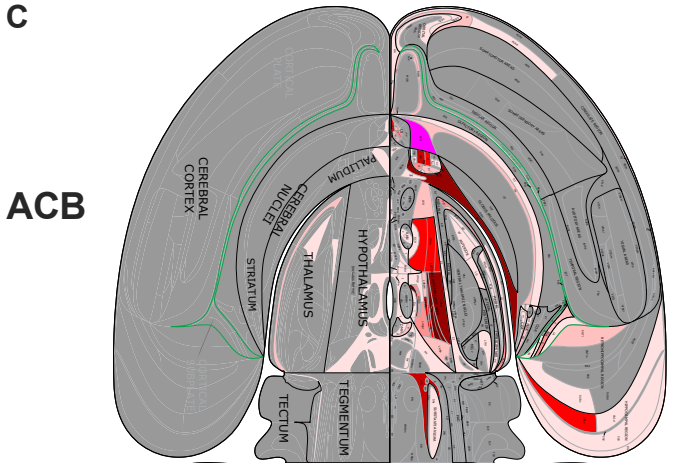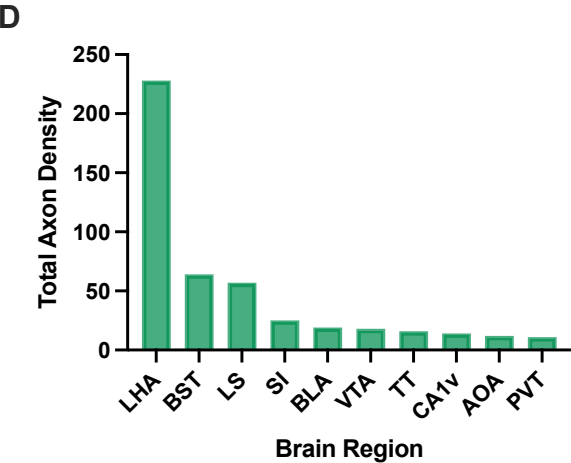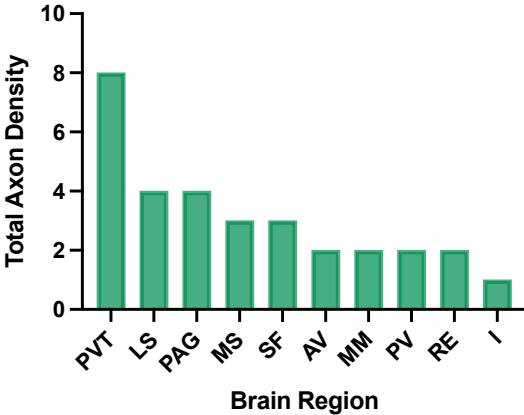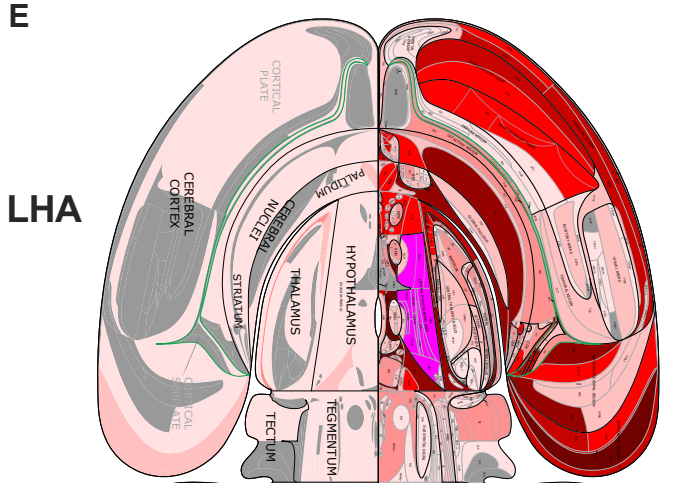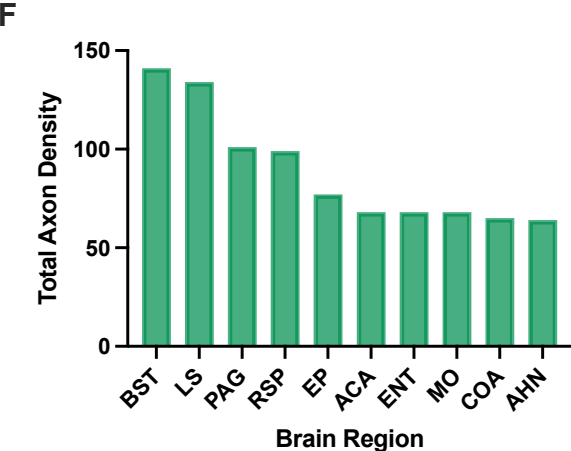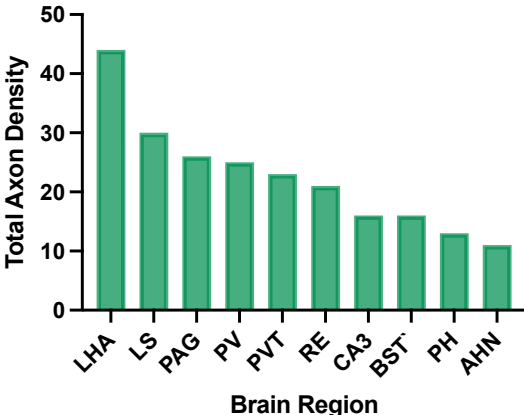

Supplement: Supplementary file 3 — Supplementary file3 (PDF 1230 KB) Supplemental Figure 3. Total axon density for anterograde tracing of 2nd order projections from CA1v pathways. A. Flatmap depicting total axon density for CA1v-mPFC anterograde tracing. B. Ipsilateral (left) and contralateral (right) graphs depicting total axon density rating in CA1v-mPFC pathway. C. Flatmap depicting total axon density for CA1v-ACB anterograde tracing. D. Ipsilateral (left) and contralateral (right) graphs depicting total axon density rating in CA1v-ACB pathway. E. Flatmap depicting total axon density for CA1v-LHA anterograde tracing. B. Ipsilateral (left) and contralateral (right) graphs depicting total axon density rating in CA1v-LHA pathway [file 429_2026_3164_MOESM3_ESM.pdf]

Supplemental Figure 4.

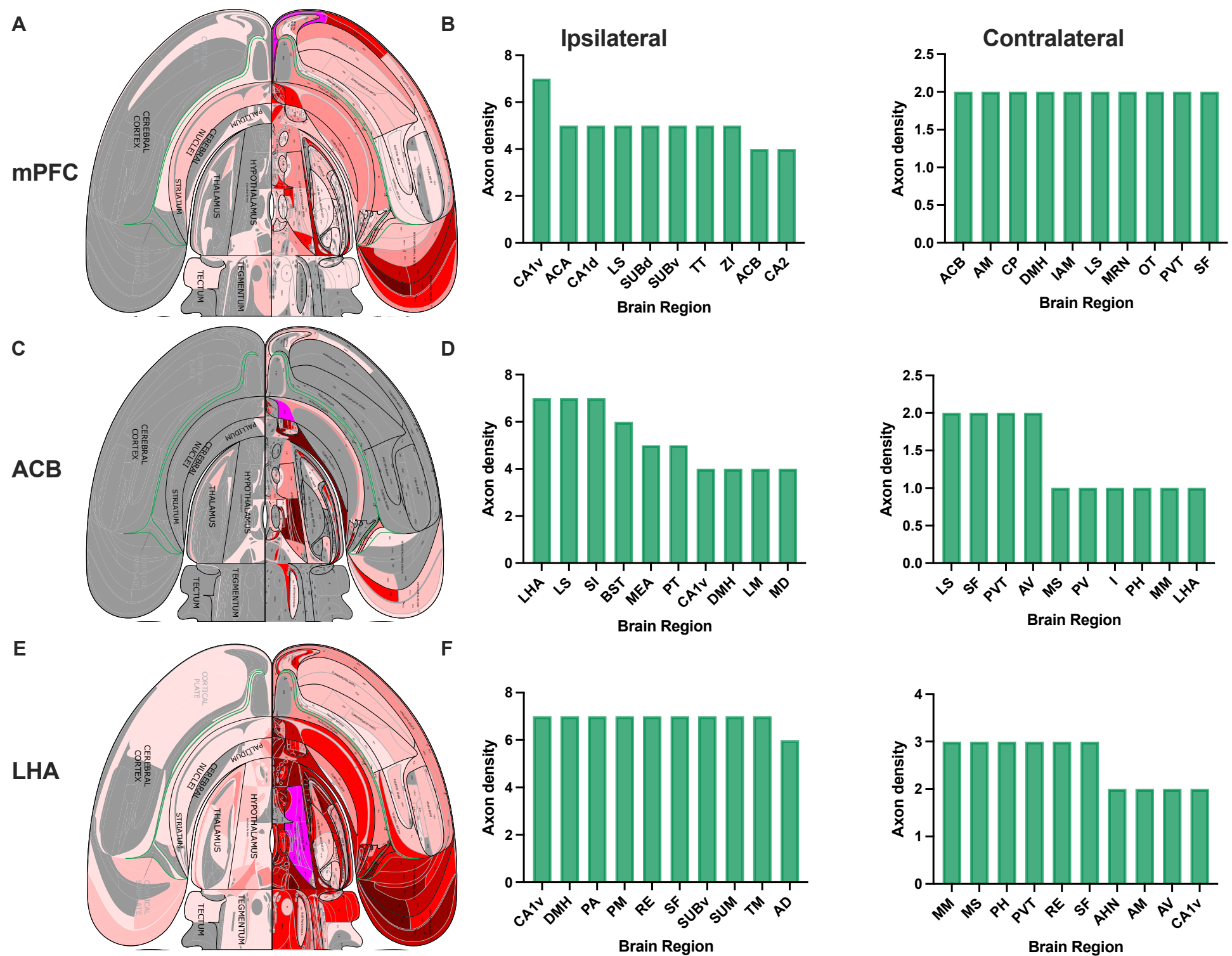

Supplement: Supplementary file 4 — Supplementary file4 (PDF 1227 KB) Supplemental Figure 4. Maximum axon density for anterograde tracing of 2nd-order projections from CA1v pathways. A. Flatmap depicting maximum axon density for CA1v-mPFC anterograde tracing. B. Ipsilateral (left) and contralateral (right) graphs depicting maximum axon density rating in CA1v-mPFC pathway. C. Flatmap depicting maximum axon density for CA1v-ACB anterograde tracing. D. Ipsilateral (left) and contralateral (right) graphs depicting maximum axon density rating in CA1v-ACB pathway. E. Flatmap depicting maximum axon density for CA1v-LHA anterograde tracing. B. Ipsilateral (left) and contralateral (right) graphs depicting maximum axon density rating in CA1v-LHA pathway [file 429_2026_3164_MOESM4_ESM.pdf]

Supplemental Figure 5.

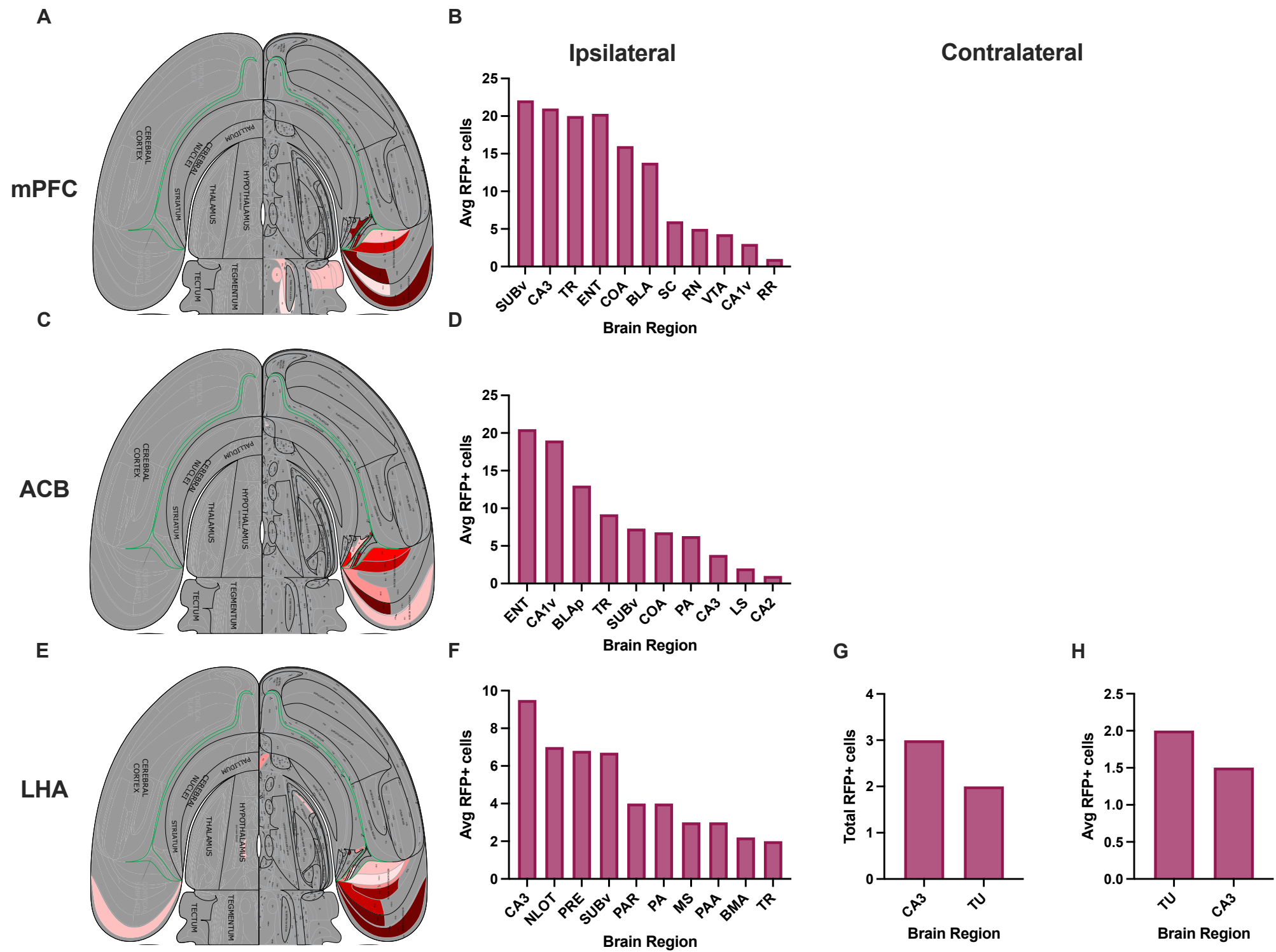

Supplement: Supplementary file 5 — Supplementary file5 (PDF 1243 KB) Supplemental Figure 5. Average RFP+ cell bodies in retrograde tracing of primary inputs to CA1v pathways. A. Flatmap depicting average RFP+ cell bodies for CA1v-mPFC retrograde tracing. B. Ipsilateral graph depicting average RFP+ cells for retrograde tracing in CA1v-mPFC pathway. C. Flatmap depicting average RFP+ cell bodies for CA1v-ACB retroograde tracing. D. Ipsilateral graph depicting average RFP+ cells for retrograde tracing in CA1v-ACB pathway. E. Flatmap depicting average RFP+ cell bodies for CA1v-LHA retrograde tracing. F. Ipsilateral graph depicting average RFP+ cells for retrograde tracing in CA1v-LHA pathway. G. Contralateral graph depicting total RFP+ cells for retrograde tracing in CA1v-LHA pathway. H. Contralateral graph depicting total RFP+ cells for retrograde tracing in CA1v-LHA pathway [file 429_2026_3164_MOESM5_ESM.pdf]
